# Life-threatening mesenchymal hamartoma of the chest wall in a neonate

**DOI:** 10.1259/bjrcr.20190004

**Published:** 2019-05-18

**Authors:** Aravind Swaminathan, Katherine Taylor, Madhavan Ramaswamy, Alistair Calder, Kieran McHugh, Nagarajan Muthialu, Quen Mok

**Affiliations:** 1 Department of Paediatric and Neonatal Intensive Care, Great Ormond Street Hospital, London,; 2 Department of Thoracic and tracheal Surgery, Great Ormond Street Hospital, London,; 3 Department of Radiology, Great Ormond Street Hospital, London,

## Abstract

Mesenchymal hamartomas of the chest wall are unusual tumours diagnosed in neonates. They mostly resolve spontaneously hence conservative management has been advocated. Some compress vital structures in the thoracic cavity or bleed warranting surgical intervention. We present a neonate with mesenchymal hamartoma of the chest wall presenting as unilateral multifocal lesions with life threatening complications. He responded well to surgical intervention and was successfully discharged.

## Introduction

Mesenchymal hamartomas (MHs) are rare chest wall tumours often manifesting at birth. They may present as innocuous mass lesions on the chest wall or with fatal compromise of intrathoracic structures.^1^ Although resection has been the traditional approach, a more conservative and observant approach has been advocated recently considering the benign and self-resolving nature of these lesions.^2^ We present a neonate with life threatening complications due to a MH of the chest wall.

## Case report

The male infant was born following spontaneous preterm labour at 34+3 weeks gestation with a birth weight of 2.35 kg. This was the mother’s first pregnancy and it had been uneventful with normal antenatal scans and protective serology. The only background of note is a maternal history of Haemophilia C. The baby had negative genetic screening and normal factor XI level. He was intubated soon after birth due to respiratory distress, and although he was extubated within 24 h, he was reintubated a few days later following an acute respiratory deterioration preceded by escalating continuous positive airway pressure and oxygen requirements.

**Figure 1.**
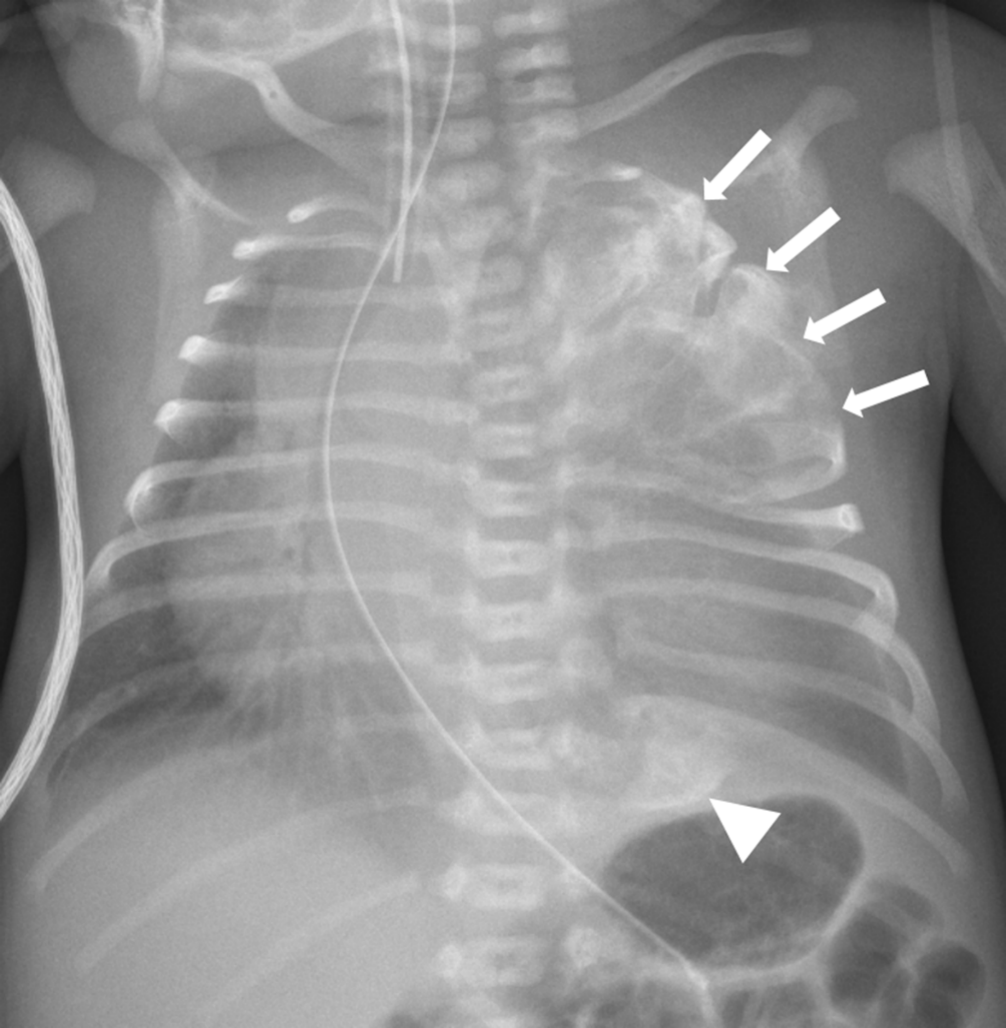
Chest radiograph at presentation. There is a large left sided upper thoracic mass, which involves and expands the left second to fifth ribs (white arrows). There is an additional lesion expanding the left 10th and 11th ribs (arrowhead). Pronounced mediastinal shift is indicated by displacement of the endotracheal tube, nasogastric tube and heart.

**Figure 2.**
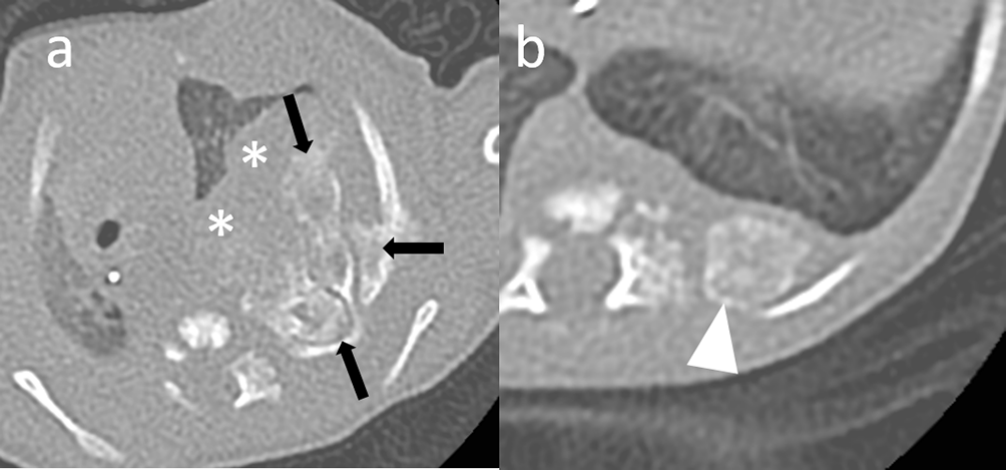
(a) Non-contrast chest CT shows marked expansion of left second to fifth ribs (arrows) with large associated soft tissue component (asterisks) and substantial mediastinal shift. (b) Additional smaller lesion involving left 10th and 11th ribs posteriorly is shown (arrow-head)

**Figure 3.**
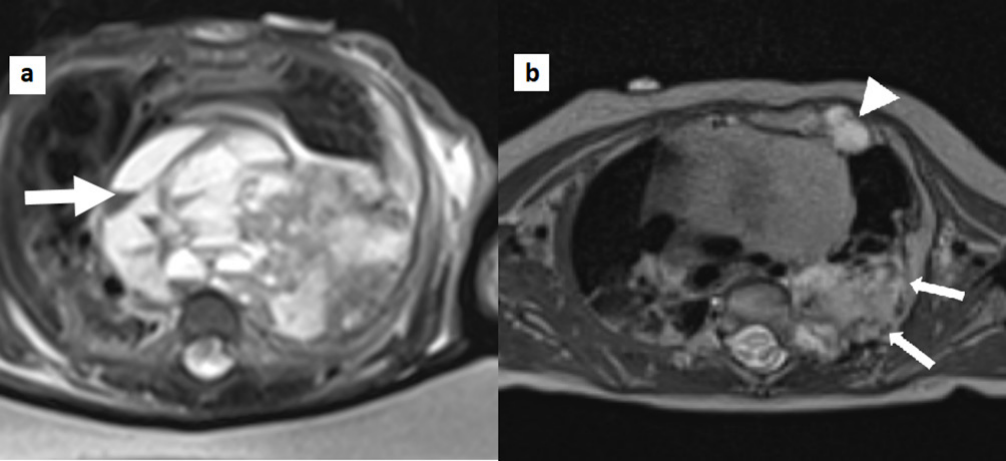
(a) Pre-operative axial thoracic MRI (T2SPC sequence). There are multiple cysts containing “fluid–fluid” levels (arrow), consistent with a large haemorrhagic cystic component. (b) 4 months post-operative axial thoracic MRI (T2SPC sequence). Mass effect has substantially resolved. There is residual disease in the left posterior ribs (arrows), in addition to the unresected left parasternal lesion (arrowhead).

Shortly after birth he was noted to have an abnormal bony prominence of his chest wall. Chest radiograph (Figure 1) showed a left-sided large upper chest wall mass causing expansion and destruction of the upper ribs, with severe tracheobronchial narrowing and mediastinal shift to the right. CT thorax (Figure 2) showed an osseous expansion of the left second to fifth ribs with a 5.8-cm sized soft tissue component, displacing the heart to the right and compressing the distal trachea and left main bronchus. Further osseous expansions were seen affecting the left 10th and 11th ribs posteriorly. These changes were suggestive of MH of the chest wall. MRI thorax (Figure 3a) showed a lobulated mass in the posterior left hemithorax, crossing the midline and returning heterogeneous signal with solid and cystic components. Some of the central cysts returned high signal on *T*_1_ weighted images with layering and fluid–fluid levels indicating internal haemorrhage. The lesion size was 4 cm × 6.5 cm × 7.3 cm displacing the heart to the right, almost contacting the chest wall. There was a significant mass effect with deviation of the trachea approximately 2.5 cm to the right with severe compression, particularly at the level of the carina and involving the bronchi. The left second to fifth ribs were abnormal and inseparable from the mass. Further lesions were noted on left 10th and 11th ribs posteriorly and left 3rd and 4th ribs anteriorly. There were concerns about the possibilities of tracheobronchomalacia and left lung hypoplasia. An echocardiogram showed a structurally normal heart but with severe dextroposition and compressed left ventricle.

**Figure 4.**
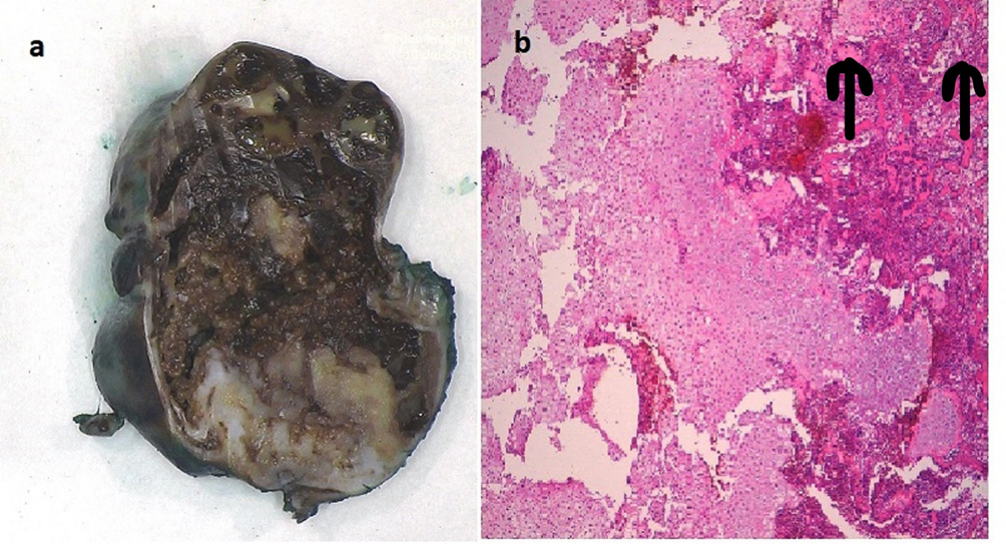
(a) Biopsy specimen: The image shows an encapsulated and nodular mass, the cut surface of which is friable and congested/haemorrhagic. The lower part of the field shows a more solid appearance, the white glistening areas representing cartilage. (b) The micrograph shows largely cellular hyaline cartilage with transition to trabecular bone (arrows in top right corner). The areas of cartilage are irregular and cellular, but the chondrocytes are not pleomorphic.

The baby was intubated, ventilated and monitored in the neonatal intensive care unit. Episodes of bradycardia and desaturation were noted when his head was turned, likely due to dynamic airway compression. Two of these episodes required cardiopulmonary resuscitation for less than a minute each time. Ultrasound-guided biopsy with aspiration of the mass and drainage of pleural effusion did not improve his symptoms. The parents were counselled that surgery entailed a high risk of catastrophic bleeding and mortality, and risk of ongoing respiratory distress with unknown development of the underlying lungs which could be hypoplastic. He underwent surgical excision of the mass along with two ribs, followed by chest wall reconstruction. The second small benign mass at 10th and 11th ribs was left behind. Histopathology of excised lesion confirmed the diagnosis as chest wall MH (Figure 4). He recovered well, was extubated after 4 days and discharged 16 days later, having established breast feeds with no respiratory compromise. When reviewed 3 months after discharge, the child was noted to be healthy. Repeat MRI (Figure 3b) revealed that the bulk of the mass had been removed with no mediastinal shift and small areas of residual tissue noted at the original sites of the non-operated lesions.

### Comment

MH of the chest wall is a rare entity, occurring with an incidence of one in a million.^3^ Diagnosis is usually made at birth but may be antenatal,^4^ or in later childhood, with one adult diagnosed and operated at 60 years of age. Clinically presenting as mass lesions on the chest wall with or without respiratory symptoms, they are not true neoplasms. These self-limiting tumours grow in early infancy and then regress after a plateau phase. Involution subsequently follows due to vascular compromise of the lesion. This natural course supports conservative management in many children, where the pressure effects are not life-threatening.^3^ Although the lesion is benign it can enlarge progressively and cause significant destruction to surrounding structures causing distortion and displacement of airways, lungs, heart, major vessels or erode ribs, as in this case. Radiologically, they present typically as a large expansile solid to cystic intrathoracic soft tissue mass arising from central position of ribs with destruction of adjacent ribs and secondary changes evident in other ribs and vertebrae. In our experience rib involvement and expansion, to a lesser degree of ipsilateral ribs remote from the major mass lesion as in this case, is commonly seen.

A unilateral multifocal mass lesion, as seen in this child, has been described in only five children in the literature to the best of our knowledge.^5^ Histopathologically, it is characteristically a solitary well-circumscribed lesion arising from ribs with cystic areas composed of mucoid and bloody contents and solid areas comprising cartilage, collagen and bone.^6^ Histology is helpful to rule out Ewing’s sarcoma or peripheral neuroectodermal tumour which mimic MH in presentation. Surgical resection is offered in children with life-threatening symptoms. Surgery can be partial resection, with good recovery or a total resection of involved chest wall. Radiofrequency thermoablation has been known to offer benefit when surgery might be too risky or radical.^7^

## Conclusion

MHs are rare chest wall tumours usually diagnosed by clinical and radiological manifestations and confirmed histopathologically. Considering the benign self-limiting nature of the lesion a conservative approach is generally adopted. However, in life-threatening situations with compression of vital intrathoracic structures, surgery becomes necessary and was live-saving in this child.

## Learning points

1. Mesenchymal hamartomas of the chest wall in neonates can cause life-threatening clinical features secondary to pressure effects on vital intrathoracic structures.
2. Imaging by MRI/CT provides valuable diagnostic information in mesenchymal hamartomas of the chest wall
3. Unilateral multifocal lesions are uncommon but do occur.
4. Surgery is life-saving when these tumours cause life-threatening features.
